# Malnutrition management in oncology: An expert view on controversial issues and future perspectives

**DOI:** 10.3389/fonc.2022.910770

**Published:** 2022-10-05

**Authors:** Paolo Bossi, Raffaele De Luca, Oriana Ciani, Elisa D’Angelo, Riccardo Caccialanza

**Affiliations:** ^1^ Department of Medical and Surgical Specialties, Radiological Sciences and Public Health, Università degli Studi di Brescia, Brescia, Italy; ^2^ Department of Surgical Oncology, Istituto di Ricovero e Cura a Carattere Scientifico (IRCCS) Istituto Tumori “Giovanni Paolo II”, Bari, Italy; ^3^ Centre for Research on Health and Social Care Management (CERGAS), Scuola di Direzione Aziendale (SDA) Bocconi School of Management, Bocconi University, Milan, Italy; ^4^ Radiotherapy Unit, Department of Oncology and Hematology, University Hospital of Modena, Modena, Italy; ^5^ Clinical Nutrition and Dietetics Unit, Fondazione Istituto di Ricovero e Cura a Carattere Scientifico (IRCCS) Policlinico San Matteo, Pavia, Italy

**Keywords:** clinical nutrition, ERAS® (Enhanced Recovery After Surgery), malnutrition, gastrointestinal (GI) cancer, head and neck (H&N) cancer

## Abstract

Cancer and anticancer treatments can lead to several negative side effects, including malnutrition. Despite the recognized need for adequate nutritional support in cancer patients, in daily clinical practice, nutrition is still not considered one of the first aspects to be considered. Malnutrition negatively affects the clinical outcomes, treatment response, and overall survival of cancer patients. In this study, three of the most controversial issues related to malnutrition, which emerged during an Italian Consensus Conference, were addressed specifically for patients with head and neck as well as gastrointestinal cancer. The timing of nutritional evaluation and intervention, extension of the Enhanced Recovery after Surgery (ERAS^®^) protocols, and cost-effectiveness of nutritional interventions have been considered. This study aimed to illustrate the state-of-the art of each issue and hypothesize future perspectives and actions to be taken, trying to suggest a new nutritional management model for cancer patients in Italy that overcomes the critical issues encountered. Of note, the timely diagnosis of nutritional issue appears to be essential to ensure the correct management of malnourished cancer patients as well as those who are at high risk of malnutrition. Standardized protocols, screening tests, and the inclusion of nutritional parameters in patient medical records would help to achieve good clinical outcomes. Finally, appropriate nutritional support is also associated with cost savings, and it seems necessary to promote its clinical and economic value to obtain improvements in both outcomes and management costs.

## 1 Introduction

Malnutrition in cancer is the result of a combination of metabolic dysregulation and anorexia, caused by the tumor itself or by its treatment (1). It negative impacts the clinical outcomes and mortality risk of cancer patients (1–7). Malnutrition is associated with a lower tolerance to anticancer treatments due to increased toxicity, a lower compliance, and a reduced response to treatments (8, 9)—increased complication rates, poor postoperative outcomes, longer hospitalization, and a poor quality of life (10–12). In particular, cancer patients have to face not only an impaired physical function but also a great deterioration in their health-related quality of life, in terms of psychological, cognitive, social, and emotional functions (10, 13–15).

Malnutrition may affect 75% of cancer patients (16–23) with a wide range of prevalence that varies according to the tumor type and stage, treatment type, patients age, and care setting (7). Approximately 15%–50% of all cancer patients present with nutritional deficiencies at the time of diagnosis, while 43% and 9% have overt malnutrition or are at risk of malnutrition, respectively, at the first oncologic visit (21, 24). This prevalence increases during treatment, reaching up to 80% of the patients (24). Cancer-related malnutrition may account for up to 20% of cancer deaths and may lead to cachexia, a significant predictor of overall survival, characterized by unintentional weight loss, low body mass index, and reduced muscle mass (25–27).

The available evidence suggests that early clinical nutrition interventions are associated with a reduction of therapy-related toxicity, an increase in relative-dose intensity, and fewer delays in cancer treatment (3, 28–35). Furthermore, an early assessment of the patient’s nutritional status and monitoring during the whole treatment course is recommended, to improve treatment tolerance, clinical outcomes, and the quality of life (3, 36).

In 2020, an Advisory Board, which included Italian Key Opinion Leaders, was established with the aim of proposing a new and optimized nutritional management model for patients with head and neck (H&N) and gastrointestinal (GI) cancer in Italy. The Advisory Board focused on these types of cancer because it is widely recognized as the highest risk of malnutrition related to them. Indeed, supportive intervention is needed in most of these patients. Therefore, these tumors have been considered by the Advisory Board a paradigm of possible tailored nutritional interventions (7, 17–21, 37–50). The Advisory Board performed a state-of-the-art analysis and identified the main critical issues regarding the clinical phases and the potential improvement actions that were required. An interregional Consensus Conference involving a multidisciplinary audience of stakeholders was then organized to reach a consensus on the priorities, to recommended action plans at a national level, and to define a nutritional management model for cancer patients in Italy.

Three of the most controversial issues from the themes that emerged during the Advisory Board meeting and Consensus Conference were the timing of nutritional evaluation and intervention, extension of the Enhanced Recovery after Surgery—ERAS^®^ protocols, and cost-effectiveness of nutritional interventions. The aims of this paper were to illustrate the state of the art of each issue, to hypothesize future perspectives, and to determine the actions to be taken.

## 2 Controversial issues in clinical nutrition in oncology

### 2.1 Timing of the nutritional evaluation and intervention

Historically, nutritional intervention occurred only in oncological patients in advanced stages of the disease, as part of a palliative treatment regimen (32). However, the efficacy of the nutritional support is linked strictly to the timing of the intervention, with the greatest efficacy being obtained with an early approach (7). Nevertheless, an early nutritional evaluation is not routinely performed (<50% of patients) (51) with a great part of patients not being identified as at risk or malnourished at the time of cancer diagnosis. Almost 65% of patients remain without any nutritional intervention (18, 49).

The detection of malnutrition and cachexia at an early stage may prevent treatment interruption, leading to higher completion rates of treatment cycles, a better tolerability of therapy, and improved outcomes (32, 52). It is recommended that a nutritional assessment be performed at any step in the oncologic pathway, with a periodical follow-up and re-evaluations of the nutritional status (53, 54) (**Table 1**).

**Table 1:** Nutritional approaches recommended by guidelines and scientific societies.

Patients to be monitored	Guideline recommendations (2, 3, 55–57)	Healthcare professional involved
**Screening and monitoring**		
All patients at the first oncologic visit	It should be performed using validated tools (NRS 2022, MUST, MST; MNA, PG-SGA) upon diagnosis and at each outpatient/follow-up visit and within 48 h of hospital admission (57)	Nurse
**Diagnosis and intervention**		
Step I: Unintentional weight loss = 0%–5% over the last 30 days	Nutritional counseling (3, 55)	Dietician
Step II: Weight loss >5% or condition associated with nutritional risk	ONS or EN (2, 3, 55, 56)	Dietician (ONS) or clinical nutritionist (EN)
Step III: Oral feeding <60% of the requirement for >1–2 weeks. Pay attention to detect the onset of dysphagia, in particular in patients with H&N undergoing radiotherapy (56)	Artificial nutrition (preferably EN) (56)	Nutritional counseling

Nutritional interventions have positive effects even in patients with a normal nutritional status.

Ho et al. (58) reported that early counseling was associated with lower median body weight change, a lower incompletion rate of planned radiotherapy, and a higher 1-year survival rate, compared with both late and no nutritional counseling. In a pragmatic randomized controlled-trial conducted in 159 H&N cancer patients undergoing radiotherapy (RT) and chemotherapy (CT) and receiving nutritional counseling, the systematic use of oral nutritional supplementation (ONS) from the start of the anticancer treatments resulted in improved weight maintenance, increased protein-calorie intake, improved quality of life, and better treatment tolerance (30).

A recent comprehensive review of the literature from the last decade (59) on the role of nutrition in the different treatment phases of gastric and esophagogastric carcinoma, concluded that, irrespective of the treatment phase, an early nutritional screening and a strict re-evaluation time is recommended, including in non-malnourished patients. The efficacy of an early nutritional intervention was also highlighted in the study of Lu et al. (60), in which patients with metastatic esophagogastric cancer, who had received early interdisciplinary supportive care, provided by a multidisciplinary team, showed improved overall survival, compared to that of patients in the standard oncologic care-alone group (14.8 vs. 11.9 months).

In patients with esophageal cancer at a high risk of malnutrition, pre-operative weight loss ≥10% was associated with a higher risk of 1-year mortality regardless of the tumor stage, age, gender, and adjuvant treatment. In these patients, early nutrition support, defined as oral or enteral nutrition supplementation during neoadjuvant treatment, was associated with less weight loss at 12 months after surgery (61). In colorectal cancer patients undergoing surgery, who had a normal nutritional status and high risk of malnutrition assessed by measuring body composition, early peripheral parenteral nutrition led to a reduction of 15.4% of postoperative complications (62).

A timely diagnosis of nutritional problems is essential to ensure the correct management of malnourished cancer patients. Therefore, it is important to define standardized protocols that provide for nutritional screening to be performed upon diagnosis by an adequately trained physician or health worker (2, 5) and the nutritional re-evaluation to be performed according to scheduled times (2, 3, 55–57). These clinical pathways would identify any variations, the impact of treatments on the nutritional status, in order to tailor patients’ therapies. The reassessment should be performed at each access time during the treatment phase, during every follow-up visit, and in cases of worsening, under the supervision of the case manager.

Screening tests are also recommended: the Malnutrition Universal Screening Tool (MUST), the Nutritional Risk Screening 2002 (NRS-2020), or the Mini Nutritional Assessment (MNA) for the early detection of malnutrition, anorexia, sarcopenia, and cachexia (3, 4, 55–57). The implementation of training courses for the medical and nursing staff on the execution and interpretation of these tests must be endorsed. Furthermore, the inclusion of nutritional parameters in the patient medical record may help identify patients who need counseling or nutritional interventions according to guidelines recommendations (2, 3, 55–57) (**Table 1**).

In addition, interestingly, the beneficial effects of nutritional supplementations may be improved by monitoring and optimizing the adherence to the dietary interventions. However, as highlighted by Faria et al. (63), who conducted a scoping review to explore the adherence to nutritional interventions in H&N cancer patients, there is a lack of evidence in this field. Available data are highly heterogeneous in terms of the definition of adherence as well as the timing and method of the assessment. Further studies are needed to assess the adherence to nutritional interventions and to identify strategies for improvement.

#### 2.1.1 Therapeutic role of calorie restriction and metabolic modulation

Nutritional interventions in cancer patients are not only effective in preventing malnutrition but they may also play an active role in the management of some types of cancer.

For example, calorie restriction interventions in the oncologic setting, based on the assumption that starvation-induced autophagy may sensitize cancer cells to chemotherapy by lowering side effects, are gaining attention recently. This assumption has been confirmed by several preclinical and preliminary clinical studies (64–69). In details, Longo’s group has shown that short-term starvation may promote an effect knows as “differential stress resistance” by selectively sensitizing different cancer cells to chemotherapeutic agents while increasing the resistance of healthy cells (67–69). In normal cells, the reduction of circulating IGF-1 and glucose levels caused by starvation can induce decreased proliferation and an increase of maintenance and repair pathways, which leads to resistance to anticancer therapies and the consequent attenuation of chemotherapy side effects (67–69). On the contrary, cancer cells showed a low fasting adaptation and continue to proliferate at a high rate, even during caloric reduction. This results in an enhanced sensitization of cancer cells to chemotherapy-induced apoptosis and an increased efficacy of treatments (67–69).

In patients who may not tolerate caloric restriction, the use of agents called caloric restriction mimetics, which mimic the caloric/energic restriction condition while allowing an adequate nutritional intake, may represent a valid alternative, but further studies are needed (69). Although, when tested under strict protocols, the preliminary clinical results are promising (69), due to the lack of solid clinical evidence, fasting and fasting-mimicking diets during active treatment are still not recommended, particularly in cancer types associated with a high risk of malnutrition as H&N, GI, and colorectal cancer. In conclusion, the idea that starving cancer cells may help increase the activity of chemotherapeutic agents is currently unclear. Prospective well-designed randomized trials on caloric restriction or caloric restriction mimetics are missing (70).

Conversely, an intriguing example of metabolic modulation as therapy comes from the management of triple-negative breast cancer (TNBC), an aggressive subtype of breast cancer. Unlike other subtypes of breast cancer (i.e., HER2+), no target therapies are available for TNBC, which is mainly treated with chemotherapy and associated with poor outcomes (71). For this reason, other therapeutic targets have been investigated, including cellular metabolism. In detail, the metabolism of a cell is heavily influenced by nutrition because micro- and macronutrients are involved in hundreds of biochemical reactions of cellular metabolism and cancer cells present altered metabolic pathways that could serve as a therapeutic target in TNBC therapies (72). Although studies in this fields are still in the early stages, the review of Wiggs et al. (72) identified potential metabolic targets in TNBC cells (i.e., glycolysis, fatty acid metabolism, autophagy, and oxidative stress-related metabolism) and nutrients and nutraceuticals that have shown to interfere with them. More studies are needed to better understand the actual role of these agents in TNBC treatment.

### 2.2 Extension of the ERAS^®^ protocol in the neoadjuvant setting

Surgery is the gold standard for GI cancer treatment in potentially curable patients (73). The surgical outcome does not depend exclusively on the surgical technical skill, and malnutrition is one of the major risk factors in surgery. In patients undergoing major cancer surgery, the evaluation of the nutritional status and the prevention and treatment of malnutrition are essential (7). Perioperative nutritional support has demonstrated efficacy in decreasing non-infectious and infectious complications and reducing the length of hospitalization (74).

The ERAS^®^ program comprises a multidisciplinary approach including the preoperative, perioperative, and postoperative phase. ERAS^®^ includes, among others, preoperative nutritional screening, to detect overt or subtle malnutrition, improving the nutritional status and the correction of specific deficits. Within this setting, perioperative immunonutrition (with arginine, omega-3 fatty acids, and RNA) has been shown to reduce postoperative complications, the length of stay, and healthcare costs significantly (75, 76). Furthermore, postoperative nutritional interventions impact on the early resumption of a normal oral diet as well as illness management and recovery outcomes after major surgery (71–81). Prehabilitation, a process in the continuum of care between the time of diagnosis and the beginning of treatment (i.e., surgery, chemotherapy, and radiotherapy) is recommended in the ERAS^®^ approach. It includes the physical, nutritional, and psychological assessments that establish a baseline functional level, identify impairments, and provide interventions that promote physical and psychological health to reduce the incidence and/or severity of future impairments (82).

In patients undergoing H&N, esophagogastric, pancreatic, and colorectal cancer surgery, the ERAS^®^ approach has been shown to reduce the surgical distress response and complication rate, to improve recovery, shorten the postoperative length of stay, and reduce hospital costs (76, 83–90).

Despite the strong evidence with regard to the safety and effectiveness of the ERAS^®^ program in different surgical settings, only few studies have focused on patients who received neoadjuvant chemotherapy (85). Nevertheless, according to the Associazione Italiana di Oncologia Medica (AIOM), the European Society for Medical Oncology (ESMO), and the National Comprehensive Cancer Network^®^ (NCCN) guidelines, neoadjuvant chemotherapy is an established treatment that improves oncological outcomes in locally advanced esophagogastric, pancreatic, and rectal cancer (91–98).

#### 2.2.1 Esophagogastric cancers

A nutritional assessment before an esophagectomy and gastrectomy is mandatory according to the ERAS^®^ guidelines (81). Pre-operative nutritional assessments, treatments and interventions are key components of this pathway with a strong recommendation grade, but with a low level of evidence. Nonetheless, an optimal nutritional approach in patients undergoing neoadjuvant chemotherapy is still lacking (99).

A small retrospective study (100) of 22 patients who were planning to undergo neoadjuvant chemotherapy for esophageal cancer, demonstrated encouraging results: the patients included in a structured pre-habilitation program, which encompassed tailored nutritional counseling, psychological support and physical exercise, had a lower weight loss (3.0% vs. 4.4%; *P* = 0.05) and readmission rate at 30 and 90-days (0.0% vs. 18.2%; *P* = 0.14 and 18.2% vs. 27.3%; *P* = 0.6, respectively) compared to the control group.

Zhao et al. (85) showed that in patients who received neoadjuvant chemotherapy for locally advanced gastric cancer, the involvement in the ERAS^®^ program, was associated with a lower post-operative complication rate (9.3% vs. 11.5%, *P* = 0.700) and a shorter post-operative length of stay (5.9 ± 5.6 vs. 8.1 ± 5.3, *P* = 0.037), compared with patients who received standard care.

#### 2.2.2 Pancreatic cancer

Pancreatic cancer is related to > 80% of the malnutrition cases, therefore in this setting early detection and prevention are the main challenges.

Several authors and scientific societies such as the ERAS, European Cancer Organization Essential Requirements for Quality Cancer Care, European Society for Clinical Nutrition and Metabolism (ESPEN) (2, 101–104) have published recommendations and guidelines to define the best management practices for pancreatic cancer patients, including the presence of a nutritionist in the extended multidisciplinary team and pre- and perioperative nutritional care for patients undergoing surgery.

Neoadjuvant chemotherapy has become the standard of care among pancreatic ductal adenocarcinoma patients with borderline resectable, locally advanced, and selected resectable disease with a feasibility of surgical resection of > 30%. In the ERAS era, prehabilitation may have been the crucial phase in which the nutritional assessment and therapy might have improved the nutritional status in preparation for the metabolic stress of surgical trauma, as reported in the randomized controlled trial by Ausania et al. (105) in which prehabilitation was associated with a lower rate of delayed gastric emptying (5.6% vs. 40.9%; *P* = 0.01) and a lower clinically relevant pancreatic fistula rate (11.1% vs. 27.3%; *P* = n.s.). The limitation of this study was the short prehabilitation time in which the patients received only 7 days of prehabilitation. In this context, Okumura et al. (106) suggested that although the ideal period of preoperative nutritional and exercise therapeutic protocols has not yet been established, at least one month before surgery is required to improve nutritional status in pancreatic cancer. However, routine nutritional screening within the ERAS^®^ programs is only implemented partially, probably due to an insufficient awareness of the nutritional features among the health professionals (54), a lack of structured collaboration between the surgeons and clinical nutrition specialists, and the absence of dedicated resources (107).

#### 2.2.3 Colorectal cancer

For the management of colorectal cancer patients, Beets et al. (108) highlighted the importance of the presence in the “extended” multidisciplinary team. Indeed dedicated and qualified nutritionists in the pre-, peri-, and postoperative settings, and during the neoadjuvant and adjuvant treatment of advanced tumors are required. The last ERAS^®^ guidelines in colorectal cancer (77) considered prehabilitation as a new item, particularly useful for patients with cardiopulmonary comorbidity and those with advanced tumors who underwent neoadjuvant chemotherapy or radiochemotherapy.

After neoadjuvant radio-chemotherapy in locally advanced rectal cancer, the timing for recovery before surgery ranges from 4–6 to 10–12 weeks A structured preparation for surgery for several weeks is very uncommon. Thereafter, this period may be “exploited” much more and used for conditioning in a prehabilitation program (109). The first randomized controlled trial conducted in this setting (110) showed the feasibility of prehabilitation in rectal cancer patients who had undergone neoadjuvant chemotherapy, based only on a 17-week physical exercise program before surgery; however, nutritional screening and prehabilitation had not been considered.

From this point of view nutritional prehabilitation, as recommended by ERAS^®^ guidelines, offers an opportunity for recovery after neoadjuvant chemotherapy and before surgery to improve the surgical outcomes and show a potential improvement in the oncological results. However, few studies have been focused on this phase and there is a low level of evidence present, while conversely, the level of recommendation is strong.

More broadly, the ERAS^®^ protocol represents the best approach for the evaluation and management of nutritional problems in cancer patients undergoing surgery and should be used in all departments of surgical oncology. Furthermore, this pathway and the use of immunonutrition allows for an optimal management of the post-operative process and a reduction in hospitalization and resource consumption (2, 77, 87), although it requires logistical and organizational efforts. It is therefore desirable to promote the formation of multidisciplinary teams, which are essential for the implementation of the ERAS^®^ program and must include surgeons, anesthetists, nurses, dieticians, pharmacists, physiotherapists, clinical nutritionists, and/or dieticians.

### 2.3 Cost-effectiveness

Malnutrition has an impact not only on clinical outcomes but also healthcare expenditure, as reported in the UK where the expenditure associated with malnutrition in the 2-year period 2011–2012 was approximately three times higher than that associated with a normal- nutritional-status patient and was equal to 19.6 billion pounds, of which 15 billion was due to healthcare services (111). Furthermore, it also assumed that if 90% of the malnourished population were identified and at least 85% of patients at a high or medium risk of malnutrition were treated according to the guidelines, a savings of £324,000–£432,000/100,000 inhabitants may be achieved in the face of an investment of £119,000–£145,000/100,000 inhabitants.

A Spanish study (112) showed that if only one-third of the patients who develop malnutrition during hospitalization received a nutritional intervention, it could result in an increase in the length of stay of approximately 7 days and an additional expenditure of 6,000 €/patient.

Cost-effectiveness analysis (113) based on the results of the above-mentioned trial by Cereda et al. (30) showed that in patients with newly diagnosed H&N cancer who were candidates for RT and/or CT, the main driver of costs was the direct cost of nutritional interventions in the group who received nutritional counseling and ONS and hospitalization costs in the group who received only nutritional counseling. Neither the differences between the two groups in quality-adjusted life years nor the costs were statistically significant, meaning that the addition of the costs of ONS were offset by the higher hospitalization and artificial nutrition costs in the control group.

From a US perspective, a project promoted by the American Society for Parenteral and Enteral Nutrition, estimated a cost saving, associated with nutritional services such as enteral nutrition, parenteral nutrition and ONS for high-priority therapeutic conditions (i.e., sepsis, GI cancer, hospital-acquired infections, surgical complications, and pancreatitis), equal to €580 million/year (114). In particular, the nutritional support in GI cancer patients could save $18–224 million €/year.

D’Angela et al. estimated the costs and benefits of immunonutrition (with arginine, omega-3 fatty acids, and nucleotides) in 15,227 well-nourished and malnourished patients who underwent esophagogastric, pancreatic, and colorectal resection in Italy (115). The use of perioperative enteral immunonutrition was associated with a reduction in the average cost of hospitalizations in well-nourished (€813.4/patient) and in malnourished patients (€1,249/patient), with a medium savings of €987. Therefore, immunonutrition would result in a cost saving to the Italian National Health Service since, against an annual investment of approximately €7.6 million (expenditure for the management expense of a malnourished patient), potential savings of €22.6 million were generated, with final annual savings of €15.0 million.

The lack of economic and human resources negatively impacts the management of the nutritional status of oncological patients since it limits either the presence of a clinical nutritionist and/or dietician in the multidisciplinary team and investments in the awareness and training of health professionals. The evidence presented above indicated that appropriate nutritional support is associated with cost savings and that it may be necessary to provide awareness-raising activities among the government institutions to promote the clinical and economic value of nutritional support and the importance of investing resources in this setting, and it may thus be possible to achieve either an improvement in short- and long-term clinical outcomes and a saving in management costs.

## 3 Discussion and future perspectives

Despite the recognized need for adequate nutritional support in cancer patients (3, 54), there is still little attention paid to nutrition in clinical practice. Furthermore, nutritional screening is not yet part of the standard clinical procedures, even in high-income jurisdictions. Thus, many patients do not receive adequate and timely support (28, 116) and, even when malnutrition is diagnosed, approximately 50% of the patients are not adequately treated or are not treated at all (18, 29).

This gap between the need and the actual nutritional interventions in cancer patients has also been pointed out in the 2022 updated practical recommendations of the Italian Intersociety Working Group for Nutritional Support in Cancer Patients (57). Although this group acknowledges the improvements that have occurred in Italy in the last 5 years in terms of awareness and institutional activities, it emphasizes the need for effective structural strategies and concrete actions to improve the clinical nutrition management of cancer patients (57). We agree with the recommendations of this group and believe that it is necessary to implement a new management model that will overcome the critical issues that have been encountered. Our practical recommendations to address these critical issues in the clinical nutrition management of cancer patients are summarized in **Table 2**. In particular, it may be essential to improve the management of the entire process, starting from the training and awareness of clinicians and the involvement of a clinical nutritionist in the multidisciplinary team, identifying the patients at a high risk of malnutrition, and taking prompt and appropriate action. The establishment of a multidisciplinary team is also essential for the success of the ERAS^®^ pathway, which represents the best approach for taking charge of cancer patients undergoing surgery.

**Table 2:** Summary of practical recommendations to address critical issues in the clinical nutrition management of cancer patients.

Area of interventions	Actions
Appropriateness of healthcare interventions	Improve the education of the clinicians. Raise the awareness of nutritional issues among the institutional stakeholders and payers and encourage investments to increase economic and human resources. Inform policymakers of the importance of standardizing access to ONS by all patients to improve therapeutic appropriateness and adherence.
Diagnosis	Perform nutritional assessments at any step of the oncologic pathway, with periodical follow-ups and re-evaluations of the nutritional status.
Management	Institute multidisciplinary teams. Involve a clinical nutritionist within the multidisciplinary team. Identify patients at a high risk of malnutrition and take prompt and appropriate action. Implement the ERAS^®^ pathway for every cancer patient undergoing surgery.

The lack of resources is a major obstacle for the improvement of the effective management of clinical nutrition in cancer patients. While nutritional screening has become more frequent, structured nutritional intervention is still lacking also due to resource shortages. Therefore, it is important to raise awareness among institutional stakeholders and payers and to encourage investments to increase the economic and human resources dedicated to the management of nutritional issues. It is equally essential to inform the policymakers of the importance of standardizing access to ONS by all the patients to improve therapeutic appropriateness and adherence.

By suggesting new paths and roles for those involved, it is anticipated that communication, collaboration, and coordination between the members of the multidisciplinary team as well as the improvement of the integration between the hospital and general practitioners in order to guarantee the continuity of care and the most appropriate management of all cases will be fostered.

In conclusion, although the main aim of our paper was to provide practical recommendations for improving the clinical nutrition management of cancer patients, we aspire to point out the lack of high-quality evidence to support nutritional interventions, emphasizing the need for well-conducted clinical and economic evaluations of nutritional interventions in oncology to provide evidence-based recommendations for efficient resource allocation polices in this area.

## Data availability statement

The original contributions presented in the study are included in the article/supplementary material. Further inquiries can be directed to the corresponding author.

## Author contributions

All authors contributed to the article and approved the submitted version.

## Funding

Editorial services and open access publication fees were funded by Nestlé Health Science. The funder was not involved in the study design, collection, analysis, and interpretation of data, the writing of this article, or the decision to submit it for publication.

## Acknowledgments

We would like to thank Ombretta Bandi from Seed Medical Publishers, who provided writing assistance and journal styling services.

## Conflict of interest

PB reports advisory board or conference honoraria from Merck, Sanofi-Regeneron, Merck Sharp & Dohme, Sun Pharma, Angelini, Molteni, Bristol-Myers Squibb, GSK, and Nestlé ED’A has served as scientific lecturer and/or advisory board and/or received unconditioned grants and/or participate to sponsored trials by Nestle Health Science, Merck spa, Astra Zeneca, Pfizer, and MSD. RC has served as scientific lecturer and/or consultant and/or on advisory panels for Baxter, Fresenius Kabi, BBraun Nestlé Health Science, and Nutricia and received unconditioned research grants by Nestlé Health Science

The remaining authors declare that the research was conducted in the absence of any commercial or financial relationships that could be construed as a potential conflict of interest

## Publisher’s note

All claims expressed in this article are solely those of the authors and do not necessarily represent those of their affiliated organizations, or those of the publisher, the editors and the reviewers. Any product that may be evaluated in this article, or claim that may be made by its manufacturer, is not guaranteed or endorsed by the publisher.
